# Medico-Chirurgical Transactions

**Published:** 1819-07-01

**Authors:** 


### *Medico-Chirurgical Transactions, Vol. IX. Part I.*

Art. 1.—*Case of [supposed] Inguinal Aneurism, cured after the Use of Compression.* By J. A. Albers, M. D.

A sailor applied to Mr. Prohfs, on account of a pulsating tumour in the right groin, as large as a hen's egg, and which had been more than a year coming. It had attained its present great size, however, in four weeks, after unusual exertion on board ship. He would not submit to an operation, and therefore pressure was the only resource.

"For this purpose we gave him a compressorium; which, like the old rupture bandages, consisted of a cushion fastened to a strap, which was buckled round the body. On the lower and inner side of the cushion, there was also a strap, which was fastened round the thigh by means of a buckle. The cushion itself consisted of two iron pieces: the uppermost had the form of a common cushion, and was externally covered with leather; the lower piece was round, and covered below with strong cloth, and above with leather. It was connected with the upper piece by a screw; by the operation of which, its pressure on the tumour could be increased or diminished at pleasure." P. 27.

After two months' pressure, violent pain took place in the aneurism, with œdema of the leg and thigh. He now left off the instrument, but the pain continued. The swelling now attained its maximum size—was red and inflamed, and fully as large as a goose's egg—the pulsation was also now very strong. The whole thigh was painful and cold. These symptoms disappeared by rest, and the compressorium was re-applied without much inconvenience. The aneurismal swelling now decreased, as did that of the thigh, and also the pain, so that the patient was enabled to go with the help of a stick.

"The amendment was now uninterrupted, until the month of June 1817, when no further pulsation could be perceived in the inguinal region. The swelling in the thigh, and the pains in it had also disappeared totally." P. 29.

We believe that every well-informed surgeon will concur in opinion with us, that no aneurism existed in this case. It was clearly a glandular swelling, to which a pulsation was communicated by the subjacent artery.

ART. 2.—*Some Observations on the Cure of Hydrocele, without procuring Obliteration of the Sac.* By Mr. KINDER WOOD.

Mr. Wood proposes an improvement on the radical operation for hydrocele, which is a kind of medium between the palliative and injection operations of the present time. It is by first letting out the water with a broad-shouldered lancet, by which a larger incision is made into the external covering than into the tunica vaginalis testis.

"When the tunica vaginalis was emptied of its contents, a small part presented at the internal opening. This was slightly hooked with a small dissecting hook, and a portion so brought forward through the internal incision, as to enable me to cut it away with a pair of fine scissars. The puncture was then closed, and supported with adhesive plaister. The parts were put into a bag truss, and the patient enjoined rest and a recumbent position."

The day after the evacuation the patient was walking about. There was some tension and tenderness of the scrotum, but no pain in the loins, or fever; the incision healed in an ordinary time, and the result was a complete cure in less than a fortnight, so that the man returned to his customary employment. Mr. Wood thinks that the removal of a small portion of the tunica vaginalis testis produces a more gentle and favourable inflammation of that membrane than the injection, and is therefore preferable. We should conceive that it will prove *uncertain,* in proportion to its mildness. Our author concludes with a case of a man who was apparently cured of an affection of the chest, attended with cough, large mucous expectoration, and dyspnœa, by a violent inflammation of the testicle after an operation for hydrocele, performed in the manner already described.

ART. 3.—*Experiments and Observations on the Union of fractured Bones.* By JOHN HOWSHIP, Esq.

This is a very interesting paper. Mr. H. by patient and laborious investigation, is daily developing to our view the processes by which Nature effects many of her most wonderful operations. To this Gentleman, therefore, the thanks of the profession are due, and he is hourly rising in the esteem of the medical public. We regret exceedingly that the limits of our Journal will not permit us to describe the very accurate and ingenious experiments, by which Mr. Howship came to the conclusions he has drawn.

ART. 4.—*Case of Aneurism in the Arm, cured by tying the Subclavian Artery.* By Dr. PORT, of New York.

THE tumour was situated at the upper and inner part of the arm, and was first discovered about three weeks before he came to New York; at which time it was about the size of a pullet's egg. Afterwards it enlarged considerably, and at the time Dr. Port first saw the patient, the tumour was extremely tense, and the pulsation strong in every part of it, particularly the apex. It was determined to tie the artery above the clavicle.

"An incision, commencing at the outer edge of the tendon of the mastoid muscle, was carried through the integuments about three inches in length, in a direction deviating a little from a parallel line with the clavicle. This divided the external jugular vein, the bleeding from which required a ligature for its suppression; and in proceeding with the operation, three or four arterial branches were cut, which it was also necessary to secure. The subclavian artery was then sought for immediately external to the scaleni muscles, and was easily laid bare. Passing over the artery at this place, in contact with it, were three considerable branches of nerves, running downwards towards the chest from the plexus above. These were separated, and the ligature passed under the artery with great facility by the instrument, well adapted to the purpose, invented by Drs. Parish, Hartshorne, and Hewson, of Philadelphia. On tying the ligature, all pulsation ceased in the limb; the edges of the wound were now brought together, and secured by sutures and adhesive straps, and a light covering of lint finished the dressing.

"The patient complaining of great pain in the whole course of the arm, eighty drops of tinct. opii were given to him, and he was put to bed. At the end of an hour, the pain continuing violent, forty drops more were administered; and this proving insufficient for his relief, an additional forty drops were taken at the end of another hour. P. 189.

About a week after the operation the tumour burst, and granulations subsequently sprung up, and the patient ultimately did well.—This operation is exceedingly honourable to trans-atlantic surgery.

ART. 5.—*Some Observations on one Species of Nævus Maternus; with the Case of an Infant, where the Carotid Artery was tied.* By JAMES WARDROP, Esq.

THE kind of tumour which is the subject of Mr. Wardrop's present observations, is formed under the skin, and situated in the cellular membrane between it and the subjacent muscles. It is always congenital, and though no part of the body is exempt from it, the face and neck are its principal seats. Its form is usually flattened; and i is very moveable, lying loose upon the muscles, and not adhering to the skin until it arrives at its advanced stage. The skin over this species retains its natural colour until the swelling becomes prominent, when the large vessels shine through, giving the diseased mass more or less of a purple hue. When small, it has a doughy and inelastic feel, like that of a spermatocele, with little sensibility. Its bulk may be greatly diminished by pressure or squeezing, and it becomes larger when the child cries, although unattended by distinct pulsation. When the bulk of the tumour is considerable, the blood-vessels which pass into it are usually of a very large size, rendering the removal of such a tumour by the knife extremely dangerous. The termination is very various. Sometimes the tumour is very small, and never increases in bulk; but at others, there is a progressive increase of size, but very gradual, so that the disease does not assume a serious aspect until the person be considerably advanced in life. In some instances it ulcerates, and then heals; in other cases, there are alarming hæmorrhages, which soon prove fatal.

*Case.* A child was born with a large subcutaneous nævus on the back part of the neck, the size of half an orange. On the tenth day, when Mr. W. saw it, a profuse hæmorrhage had taken place, and yet there was no diminution of the tumour. There was no distinct pulsation, but a violent throbbing was felt in the tumour, and in the arteries leading to it. Our author extirpated the tumour as expeditiously as he could, but the hæmorrhage was so excessive, that the child died under the operation. This circumstance led our author to propose the practice adopted in the following Case.

An infant was brought to London with a subcutaneous nævus on the left cheek, the base of which extended from the temple to beyond the angle of the jaw, enveloping the cartilage of the ear. A portion of the integuments was ulcerated. The skin on the rest of the tumour was covered with turgid vessels. The child was then six weeks old. The tumour was about the bulk of a small-sized orange at birth, and had been daily increasing, and there had lately been several profuse hæmorrhages. Mr. Wardrop, assisted by Mr. George Young and Mr. Travers, tied the carotid artery. The operation produced no change in the child's countenance; but, in a very few hours, there was a manifest alteration in the appearance of the tumour.

It became soft and pliable, lost its purple colour, and the tortuous veins collapsed.

"The child died on the fourteenth day after the operation, exhausted by the irritation of an ulcer, which had now involved the whole surface of an enormous tumour." P. 212.

Although Mr. Wardrop is entitled to every praise for the attempt to save the life of this child, yet we confess, that a formidable operation, superadded to a formidable disease, in an infant so exhausted as to require "milk, beef tea, brandy and opium" the very day before the carotid artery was taken up, precluded almost the hope of success.

Some general remarks and general rules on the subject of nævi close the paper; which, though somewhat prolix, and rather too systematical and studied, evince a considerable share of talent and acute observation in the author, and of which, indeed, Mr. Wardrop has often before given incontestible proofs.

### Art. 6.—*An extensive Wound from the Bite of a Shark.* By Dr. Kennedy.

A pearl-diver, in the Gulf of Manaar, was seized by a shark under water, and would have been carried off, had he not been secured by a rope. It appeared that the upper jaw of the animal had been fixed on the left side of his belly, forming a wound of nearly semi-circular shape. The abdominal and lumbar muscles were divided and turned up, exposing the colon in its passage across the belly, and several other convolutions of the small intestines. Three of the lowest ribs were laid bare; the gluteal muscles were lacerated and torn up; the tendons about the trochanter divided, laying bare that tuberosity; the vastus externus and part of the rectus muscles of the thigh were cut across. The lower jaw of the fish had been fixed in the muscles of the right side of the abdomen, forming a segment of a circle between the umbilicus, and an inch above the pubes; the teeth of this jaw, however, had only individually penetrated the muscles, without producing any division or laceration of the parts. The wound of the left side measured nineteen inches; in breadth, where the abdominal and lumbar muscles were lacerated, between four and five inches; below this, and above the trochanter, two; and at and below the trochanter, three and four inches. The resistance made by the trochanter certainly prevented that part of the body in the grasp of the shark from being taken away. All moisture was removed by a sponge from the wound; it was closed, as well as possible, by adhesive straps; after which a light dressing was applied, and a many tailed bandage thrown over the whole.

The after-treatment was equally judicious and successful; and, as Dr. Kennedy justly observes, the patient is likely "long to bear about with him, on the shores of India, a striking example of the benefit of British surgery."

Did our fair country-women consider at what an expence of human life and human health those pearly decorations are procured, with which they encircle their necks and bosoms, they would cast them off, and commit them to the ocean from whence they were dragged! To encourage that horrible fishery is to league themselves with sharks, and degrade human Nature *beneath* the level of the brute creation, for the animal only seeks his prey to satisfy the cravings of his appetite!

We have thus concluded the surgical part of this work, and though there is not so much variety of surgical facts in this as in some of the preceding volumes, chiefly because there are more medical papers, yet, as our readers must have perceived, the Miscellany is highly respectable, and supports the reputation of the Society in a dignified manner.

